# Validity Study of the “Imbalanced Energy Field” Nursing Diagnosis

**DOI:** 10.1590/0034-7167-2025-0119

**Published:** 2026-02-23

**Authors:** Camila de Souza Carneiro, Ana Cristina de Sá, Gisele Saraiva Bispo Hirano, Viviane Martins Silva, Alba Lucia Bottura Leite de Barros

**Affiliations:** I Universidade Federal de São Paulo, Hospital São Paulo. São Paulo, São Paulo, Brazil.; II Therapeutic Touch International Association. São Paulo, São Paulo, Brazil.; III Centro Universitário das Faculdades Metropolitanas Unidas. São Paulo, São Paulo, Brazil; IV Universidade Federal do Ceará. Fortaleza, Ceará, Brazil.; V Universidade Federal de São Paulo. São Paulo, São Paulo, Brazil.

**Keywords:** Nursing Diagnosis, Validation Studies, Delphi Technique, Standardized Nursing Terminology, Spiritual Therapies

## Abstract

**Objectives:**

to analyze the “Imbalanced Energy Field” (00273) nursing diagnosis content.

**Methods:**

a content validity study of a nursing diagnosis. Twelve judges assessed the relevance of the elements of the NANDA-I “Imbalanced Energy Field” nursing diagnosis and the clarity and precision of its conceptual and operational definitions. A Content Validity Index ≥0.80 was considered adequate.

**Results:**

all definitions were analyzed by judges, in addition to the validity of the 22 defining characteristics and five related factors already existing in NANDA-I 2021–2023. The exclusion of seven defining characteristics and the modification of ten terms were suggested (Delphi Agreement Index ≥80%). Conceptual and operational definitions of these defining characteristics were constructed, validating them using a Content Validity Index ≥0.80.

**Conclusions:**

after validity, 15 defining characteristics and five related factors were deemed relevant and the suggested changes were approved and published by NANDA-I 2024-2026.

## INTRODUCTION

The nursing diagnoses (NDs) described in NANDA-I taxonomy aim to provide a scientific basis for the practice of care, directed to a therapeutic plan focused on accurate nursing interventions^(1)^.

The “Disturbed Energy Field” (00050) ND was inserted in NANDA-I in 1994^(1)^ with the definition: “Disturbance of the flow of energy that involves a person, resulting in disharmony of the body, mind and/or spirit”. In 2011, due to the lack of evidence for this diagnosis and due to its similarity with the Nursing Interventions Classification “Therapeutic Touch” (5465) intervention^(2)^, NANDA-I decided to review it based on studies of conceptual reviews, concept analysis of its diagnostic elements and a qualitative study that used online questionnaires with 426 holistic nurses who used it when applying energetic techniques^(3,4)^.

From these searches, the “Imbalanced Energy Field” (00273) ND was inserted into the NANDA-I 2018-2020 taxonomy^(1)^ and was modified in NANDA-I 2021-2023^(5)^, although it has not yet been validated for its effective applicability in clinical practice. *A priori*, it is identified that in its related factors (RFs), emotional disorders predominate, such as anxiety and excessive stress. Stress-related factors affect 90% of the world’s population and are closely linked to diseases of the circulatory system. Stress and anxiety threaten individuals’ balance and well-being, causing physical somatic changes such as gastritis, hypertension, diabetes mellitus, among other comorbidities^(6–8)^.

A previous study developed the conceptual and operational definitions for the ND defining characteristics (DCs) and RFs, because in addition to being the first stage of the diagnostic validity study, this diagnosis has the terms “energy field” and “energy flow”, which are still heavily criticized by biomedical science and needed to be conceptualized to facilitate their clinical applicability^(9)^.

## OBJECTIVES

To analyze the “Imbalanced Energy Field” diagnosis content, in order to contribute to the development of a more consistent diagnostic structure that allows better support to clinical nursing practice based on nurses’ diagnostic inference process.

## METHODS

A study was carried out to validate the content of an ND, according to the modified stages proposed by Hoskins^(10)^. According to this author, the validity process should occur in three stages: integrative review; DC and RF content validity in a study with specialists; and clinical validity of the ND DC and RF conceptual and operational definitions. The first stage consists of an integrative review to conceptually and operationally define the DCs and RFs of the “Imbalanced energy field” diagnosis^(9)^, as the present study deals with the second stage (DC and RF validity with specialists), which took place from June to October 2022.

### Sample definition

The sample was composed of nurses with academic and clinical experience with NDs and/or Integrative and Complementary Health Practices (ICHP).

Professionals were initially selected based on the minimum criteria established by the researchers, including: practical experience with NDs and/or ICHP; academic experience; and participation in research groups on the topic. Their level of expertise was defined according to their scores: a minimum score of 5 points for a junior specialist; a score between 5 and 11 points for a master specialist; a maximum score of 11 points for a senior specialist^(11)^.

The data from specialists were collected directly from the *Lattes* Platform and from the Brazilian National Council for Scientific and Technological Development Research Group Directory. The invitation, made via e-mail, included a description of the study objectives and an Informed Consent Form (ICF). After accepting and signing the ICF, the selected specialists received an electronic form with questions about their profile, sociodemographic characterization, and conceptual and operational definitions of the 22 DCs and the five RFs described in NANDA-I 2021-2023^(5)^. Evaluators assigned a value regarding the relevance of the 22 DCs and the five RFs, as well as the accuracy and clarity of their respective conceptual and operational definitions, on a Likert-type scale from 0 to 2, as follows: 0 – inadequate; 1 – little adequate; and 2 – adequate. Items that obtained agreement equal to or greater than 80% among judges were considered adequate.

The sample size of judges in content validity studies using the Delphi technique has been a major obstacle. Although there is no definition of a minimum number of specialists for the use of this technique, a minimum of ten was established for this study, as recommended in the literature^(12)^.

The invitation to participate in this stage of the research was sent by e-mail to 20 nurses, considering the difficulty previously documented in similar studies regarding the rate of return of forms filled out in a timely manner^(13)^. The sample totaled 12 specialists in the first round, 11 specialists in the second round, and ten specialists in the third round of assessment.

### Data analysis and treatment

Specialist characterization data were analyzed according to absolute (f) and relative (%) frequencies. The items of the instrument that reached a consensus among the specialists of 80% or more were considered necessary and were kept in the data collection instrument. The suggestions of the participants considered pertinent were included and sent for further judgment by the specialists. The proportion and 95% Confidence Interval (95%CI) of all diagnostic elements were calculated, in addition to their conceptual and operational definitions.

### Ethical aspects

The study was conducted in accordance with national and international ethics guidelines, and was approved by the *Universidade Federal de São Paulo* Research Ethics Committee (Certificate of Presentation for Ethical Consideration 29110620.0.0000.5505), whose opinion is attached to this submission. All judges signed an ICF, and anonymity was guaranteed.

## RESULTS

All specialists who participated in this study were nurses (12). There was a predominance of females (11), working in southeastern Brazil (five) and in the Northeast and South regions (three), respectively, with a maximum doctoral degree (12), current occupation as professors and researchers (11), exercising their professional activities in educational institutions (11).

In relation the use of the ND, nine judges stated that they use it both in clinical practice and in teaching. Of the judges, nine taught subjects related to NDs and 12 taught ICHP. As for judges’ age, seven are between 30 and 45 years old, and five between 50 and 65 years old. All specialists have professional experience with ICHP over 12 years, and eight participate in a research group on the subject.

To reach a minimum consensus of 80% for the “Imbalanced energy field” diagnosis elements content validity, three rounds were required. In addition to the level of agreement, the proportion and 95% Confidence Interval (95%CI) of the DC and RF were calculated, in addition to their conceptual and operational definitions. Table 1 describes the results of the first round.

**Table 1:** Results of specialists’ assessment in the first round of assessment of the diagnostic elements and their conceptual and operational definitions (N = 12), São Paulo, São Paulo, Brazil, 2025

Items	n	%	95%CI CD	n	%	95%CI CD	n	%	95%CI
DC - Congested energy field patterns	9	75.0	42.8 – 93.3	7	58.3	28.5 – 83.5	7	58.3	28.5 – 83.5
DC - Slow energy field patterns	9	75.0	42.8 – 93.3	6	50.0	25.3 – 74.6	6	50.0	25.3 – 74.6
DC - Fast energy field patterns	9	75.0	42.8 – 93.3	5	41.6	16.4 – 71.4	6	50.0	25.3 – 74.6
DC - Strong energy field patterns	10	83.3	50.8 – 97.0	5	41.6	16.4 – 71.4	6	50.0	25.3 – 74.6
DC - Weak energy field patterns	10	83.3	50.8 – 97.0	5	41.6	16.4 – 71.4	6	50.0	25.3 – 74.6
DC - Pulsations felt in energy flow	8	66.6	35.4 – 88.7	6	50.0	25.3 – 74.6	6	50.0	25.3 – 74.6
DC - Tumultuous energy field patterns	6	50.0	25.3 – 74.6	5	41.6	16.4 – 71.4	6	50.0	25.3 – 74.6
DC - Dissonant rhythms felt in energy flow	8	66.6	35.4 – 88.7	6	50.0	25.3 – 74.6	6	50.0	25.3 – 74.6
DC - Energy field patterns with frequency ranging from pulsatile to throbbing	7	58.3	28.5 – 83.5	5	41.6	16.4 – 71.4	6	50.0	25.3 – 74.6
DC - Random energy field patterns	7	58.3	28.5 – 83.5	5	41.6	16.4 – 71.4	6	50.0	25.3 – 74.6
DC - Irregular energy field patterns	11	91.6	59.7 – 99.5	8	66.6	35.4 – 88.7	6	50.0	25.3 – 74.6
DC - Arrhythmic energy field patterns	6	50.0	25.3 – 74.6	5	41.6	16.4 – 71.4	6	50.0	25.3 – 74.6
DC - Hot temperature differentials in energy flow	10	83.3	50.8 – 97.0	6	50.0	25.3 – 74.6	6	50.0	25.3 – 74.6
DC - Cold temperature differentials in energy flow	10	83.3	50.8 – 97.0	5	41.6	16.4 – 71.4	6	50.0	25.3 – 74.6
DC - Magnetic traction for an energy field area	11	91.6	59.7 – 99.5	5	41.6	16.4 – 71.4	6	50.0	25.3 – 74.6
DC - Energy flow energy deficit	9	75.0	42.8 – 93.3	5	41.6	16.4 – 71.4	6	50.0	25.3 – 74.6
DC - Energy flow hyperactivity	8	66.6	35.4 – 88.7	5	41.6	16.4 – 71.4	6	50.0	25.3 – 74.6
DC - Energy flow congestion	8	66.6	35.4 – 88.7	7	58.3	28.5 – 83.5	6	50.0	25.3 – 74.6
DC - Blocking of energy flow	8	66.6	35.4 – 88.7	5	41.6	16.4 – 71.4	6	50.0	25.3 – 74.6
DC - Tingling sensation in energy flow	9	75.0	42.8 – 93.3	5	41.6	16.4 – 71.4	6	50.0	25.3 – 74.6
DC - Dissonant rhythms of energy field patterns	6	50.0	25.3 – 74.6	6	50.0	25.3 – 74.6	7	58.3	28.5 – 83.5
DC - Expression of the need to recover the experience of the whole	9	75.0	42.8 – 93.3	9	75.0	42.8 – 93.3	7	58.3	28.5 – 83.5
RF - Anxiety	11	91.6	59.7 – 99.5	7	58.3	28.5 – 83.5	9	75.0	42.8 – 93.3
RF - Discomfort	12	100.0	69.8 – 100.0	10	83.3	50.8 – 97.0	8	66.6	35.4 – 88.7
RF - Interventions that disturb the energy flow or pattern	9	75.0	42.8 – 93.3	6	50.0	25.3 – 74.6	7	58.3	28.5 – 83.5
RF - Excessive stress	10	83.3	50.8 – 97.0	11	91.6	59.7 – 99.5	10	83.3	50.8 – 97.0
RF - Pain	12	100.0	69.8 – 100.0	10	83.3	50.8 – 97.0	10	83.3	50.8 – 97.0

*DC - defining characteristic; RF - related factor; CD - conceptual definition; OD - operational definition; 95%CI – 95% Confidence Interval*.

From the second round onwards, in addition to assessing the relevance of the DCs and RFs, and the clarity and accuracy of the conceptual and operational definitions of these diagnostic elements, the meanings and semantics of the terms of DCs and RFs were also analyzed. At this time, suggestions were made to change terms and even exclude some DCs. These modifications were:

The “Slow energy field patterns” DC was changed to “Slow energy flow pattern” (90.9%CI); the “Fast energy field patterns” DC was changed to “Fast energy flow pattern” (90.9%CI); the “Strong energy field patterns” DC was changed to “Increased or high energy field pattern” (100%CI); the “Weak energy field patterns” DC was changed to “Low energy field pattern” (90.9%CI); the “Asynchronous rhythms felt in energy flow” DC was changed to “Asynchronous rhythms in energy flow” (81.8%CI); the “Hot temperature differentials in energy flow” DC was changed to “Hot temperature differentials in energy field or flow” (100%CI); the “Cold temperature differentials in energy flow” DC was changed to “Cold temperature differentials in energy field or flow” (100%CI); the “Magnetic traction for an energy field area” DC was changed to “Traction for an energy field area” (90.9%CI); the “Expression of the need to recover the experience of the whole” DC was changed to “Expression of feeling disintegrated, unmotivated or maladjusted” (90.9%CI); and the “Interventions that disturb the flow or pattern” RF was changed to “Health treatments and/or procedures that disturb the energy flow or pattern” (81.8%CI).

The “Random energy field patterns” and “Tingling sensation in energy flow” DCs were excluded in the third round, considering that its concept and operationalization are already contemplated in other DCs (80%CI and 100%CI). The “Energy flow hyperactivity” DC was excluded, since the literature does not describe this event and specialists are also unaware of it (100%CI); the DC “Tumultuous energy field patterns” was excluded because its terms are synonymous with the “Irregular energy field patterns” DC (100%CI); the “Energy flow congestion” DC was excluded, because the “Congested energy field patterns” DC already identifies energy congestion (90.9%CI); the “Dissonant rhythms of energy field patterns” DC was excluded, since its terms are synonymous with the “Tumultuous energy field patterns” DC (100%CI); and the “Arrhythmic energy field patterns” DC was excluded, as energy field “arrhythmicity” is not identified in the literature or in specialists’ clinical practice (100%CI).

Table 2 details the results of the consensus among specialists, the proportion and the 95%CI of the second round, with the exception of items that were excluded from the instrument.

**Table 2:** Results of specialists’ assessment in the second round of assessment of the diagnostic elements and their conceptual and operational definitions (N = 11), São Paulo, São Paulo, Brazil, 2025

Items	n	%	95%CI CD	n	%	95%CI CD	n	%	95%CI
DC - Congested energy field patterns	10	90.9	57.1 – 99.5	10	90.9	57.1 – 99.5	10	90.9	57.1 – 99.5
DC - Slow energy flow pattern	10	90.9	57.1 – 99.5	9	81.8	47.7 – 96.7	9	81.8	47.7 – 96.7
DC - Fast energy flow pattern	10	90.9	57.1 – 99.5	9	81.8	47.7 – 96.7	9	81.8	47.7 – 96.7
DC - Increased or high energy field pattern	11	100.0	67.8 – 100.0	10	90.9	57.1 – 99.5	10	90.9	57.1 – 99.5
DC - Low energy field pattern	10	90.9	57.1 – 99.5	8	72.7	39.3 – 92.6	9	81.8	47.7 – 96.7
DC - Pulsations felt in energy flow	11	100.0	67.8 – 100.0	11	100.0	67.8 – 100.0	9	81.8	47.7 – 96.7
DC - Irregular energy field patterns	11	100.0	67.8 – 100.0	9	81.8	47.7 – 96.7	9	81.8	47.7 – 96.7
DC - Asynchronous rhythms in energy flow	9	81.8	47.7 – 96.7	9	81.8	47.7 – 96.7	7	63.6	31.6 – 87.6
DC - Energy field patterns with frequency ranging from pulsatile to throbbing	10	90.9	57.1 – 99.5	10	90.9	57.1 – 99.5	8	72.7	39.3 – 92.6
DC - Random energy field patterns	8	72.7	39.3 – 92.6	8	72.7	39.3 – 92.6	9	81.8	47.7 – 96.7
DC - Hot temperature differentials in energy field or flow	11	100.0	67.8 – 100.0	10	90.9	57.1 – 99.5	8	72.7	39.3 – 92.6
DC - Cold temperature differentials in energy field or flow	11	100.0	67.8 – 100.0	11	100.0	67.8 – 100.0	10	90.9	57.1 – 99.5
DC - Magnetic traction for an energy field area	10	90.9	57.1 – 99.5	11	100.0	67.8 – 100.0	10	90.9	57.1 – 99.5
DC - Energy flow energy deficit	10	90.9	57.1 – 99.5	9	81.8	47.7 – 96.7	8	72.7	39.3 – 92.6
DC - Blocking of energy flow	9	81.8	47.7 – 96.7	8	81.8	47.7 – 96.7	8	72.7	39.3 – 92.6
DC - Tingling sensation in energy flow	8	72.7	39.3 – 92.6	8	72.7	39.3 – 92.6	7	63.6	31.6 – 87.6
DC - Expression of feeling disintegrated, unmotivated, or maladjusted	10	90.9	57.1 – 99.5	10	90.9	57.1 – 99.5	9	81.8	47.7 – 96.7
DC - Hyperactivity of energy flow	8	72.7	39.3 – 92.6	8	72.7	39.3 – 92.6	7	63.6	31.6 – 87.6
DC - Tumultuous energy field patterns	7	63.6	31.6 – 87.6	7	63.6	31.6 – 87.6	8	72.7	39.3 – 92.6
DC - Dissonant rhythms of energy field patterns	9	81.8	47.7 – 96.7	7	63.6	31.6 – 87.6	7	63.6	31.6 – 87.6
DC - Energy flow congestion	7	63.6	31.6 – 87.6	8	72.7	39.3 – 92.6	9	81.8	47.7 – 96.7
DC - Arrhythmic energy field patterns	7	63.6	31.6 – 87.6	7	63.6	31.6 – 87.6	7	63.6	31.6 – 87.6
RF - Anxiety	9	81.8	47.7 – 96.7	10	90.9	57.1 – 99.5	11	100.0	67.8 – 100.0
RF - Discomfort	11	100.0	67.8 – 100.0				8	72.7	39.3 – 92.6
RF - Health treatments and/or procedures that disturb the energy flow or pattern	11	100.0	67.8 – 100.0	9	81.8	47.7 – 96.7	10	90.9	57.1 – 99.5
RF - Pain	11	100.0	67.8 – 100.0	10	90.9	57.1 – 99.5			

*DC - defining characteristic; RF - related factor; CD - conceptual definition; OD - operational definition; 95%CI – 95% Confidence Interval*.

The assessment of the third round of the diagnostic elements and their conceptual and operational definitions, with the exception of items that were suppressed from the final instrument, reached a consensus above 80%. They are: “Low energy field patterns” DC conceptual definition; “Asynchronous rhythms in energy flow” DC operational definition; “Energy field patterns with frequency ranging from pulsatile to throbbing” DC operational definition; “Hot temperature differentials in energy field or flow” DC operational definition; “Energy flow energy deficit” DC operational definition; “Blocking of energy flow” DC operational definition.

Due to the great extension of the conceptual and operational definitions validated by this study, only one model will be presented below (Chart 1).

**Chart 1:** Conceptual and operational definitions elaborated for the defining characteristics and related factors of the “Imbalanced Energy Field” diagnosis, São Paulo, São Paulo, Brazil, 2025

*Defining characteristics*	*Conceptual definition*	*Operational definition*
Congested energy field patterns^(3,4,9,14–18)^	Sensory perception of energy field congestion, with bulging of the field immediately prior to partial or total interruption of energy field and energy flow accompanied by sensory perception of back pressure, numbness, and/or shock.	The procedure should be explained to the participant, and then the practitioner should: (1) consciously center themselves, focusing on the participant, and thus activate a state of extended perception; (2) assess the energy field, keeping the hands at a distance between 5 and 10 cm with a margin of difference of 2 to 4 cm more or less from the patient’s body, keeping them in the tangency between the human energy field and the environmental energy field, to explore congestion in the energy field, which generates bulging, energy flow deficits, and unbalanced energy; (3) treat the affected areas and modulate the human field, to direct and balance the energy in its dimension and symmetry, with the aim of promoting free flow of energy and human field harmonization as a whole; in cases of congestion and backpressure, it may be necessary to perform, in addition to the smoothing movement, a field unblocking movement to restore flow in the area immediately after the area of congestion in the cephalopodal direction; and (4) assess the energy field and finalize treatment.
***Related factors***	***Conceptual definitions***	***Operational definitions***
Anxiety^(3,4,7,9,14–18)^	Signs and symptoms of stress; anxiety; exaggerated concerns about health, money, family, or work; tension or extreme fear of a particular object or situation; exaggerated fear of being humiliated; a constant feeling that a disaster or something very bad is going to happen; lack of control over thoughts, images, or attitudes, which repeat themselves regardless of will; dread after a very difficult situation.	Apply the Hospital Anxiety and Depression Scale to assess the level of anxiety and depression.

This research required three rounds to improve the “Imbalanced Energy Field” diagnosis elements and its conceptual and operational definitions, which were submitted to NANDA-I for its improvement and better applicability in clinical practice.

## DISCUSSION

The conceptual and operational definitions of ND DCs and RFs are essential to validate their content, as they establish observational and practical descriptions for the improvement and clinical applicability of the diagnosis^(14)^.

Furthermore, they can serve to increase the reliability and validity of clinical data related to diagnoses. Moreover, they can favor the replication of research, improve the researcher's ability to correlate the findings of previous studies, and enable the construction of indicators for choosing nursing interventions and assessing outcomes^(15–19)^.

The “Slow energy field patterns”, “Fast energy field patterns”, “Hot temperature differentials in energy flow”, and “Cold temperature differentials in energy flow” DCs were changed, since the researchers and specialists during the validity stages identified that the terms “energy field” and “energy flow” were described as synonyms in DCs. However, their concepts, psychic, and energetic constitutions are different. The “Random energy field patterns”, “Tingling sensation in energy flow”, “Energy flow hyperactivity”, “Tumultuous energy field patterns”, “Irregular energy field patterns”, “Energy flow congestion”, “Dissonant rhythms of energy field patterns”, and “Arrhythmic energy field patterns” DCs were also excluded because specialists identified that they had no evidence in the literature and did not use them in clinical practice, and also the terms were repetitive and did not effectively represent clinical indicators. The “Interventions that disturb the energy flow or pattern” RF was changed to “Treatments and/or health procedures that disturb the energy flow or pattern”, since the modified terms are clearer and more precise. The content validity of this ND was accurate so that it can really be used in clinical practice safely and without doubts as to the meanings and relevance of its elements. The human energy field or aura is the part of the universal energy associated with the physical body, and the chakras are the vortices that move and metabolize the energy from the universal energy field to the human energy field. In other words, the chakras are openings through which the flow of energy from the aura enters and leaves and diseases are usually caused by energy flow obstruction or interruption^(20–24)^.

It is well known that tension, bad mood, negative thoughts, and improper decisions in everyday life lead individuals to a state of physical and emotional overload, resulting in fatigue and other imbalances that can exhaust organic functions. The human body, being highly efficient and having considerable functional reserve, normally adapts to these overloads. However, when stress becomes excessive, such adaptation ceases to occur, generating somatization of diseases in areas of the body that are already vulnerable due to hereditary factors or genetic predispositions. Examples of these problems include gastritis, colitis, insomnia, headache, dizziness, recurrent colds, neck or lower back pain, diarrhea, and palpitations^(20,25)^.

Still from these perspectives, it was also suggested to the NANDA-I taxonomy that there should be a change in some terms in the “Imbalanced Energy Field” diagnosis definition for semantic adequacy, as well as adequacy to the concept of human energy field (human electromagnetic field) that differs from energy flow, as described above. Therefore, the definition of the diagnosis under analysis was changed from “Disruption in the vital flow of human energy, which is usually a single, dynamic, creative and nonlinear continuous whole” to “An interruption in the human electromagnetic field that is usually a continuous and unique, dynamic, creative and nonlinear whole”.

In addition, it has been suggested that the “Imbalanced Energy Field” diagnosis domain and class be changed due to humanistic and holistic theory foundations^(25–27)^ and collective health paradigms. Such suggestions were forwarded to the ND committee of NANDA-I, which led to the change in the allocation of the “Imbalanced Energy Field” diagnosis in another domain and class in the 2024-2026 edition (Domain 1 - Health Promotion; and Class 1 - Health Perception)^(26)^.

Based on the broad conception of the health-disease process and its determinants, health promotion proposes the articulation of technical and popular knowledge, and the alliance of institutional, community, public, and private resources, favoring quality of life, solidarity, equity, democracy, citizenship and integration of individuals in therapies with autonomy and empowerment^(27)^.

Nurses assess information from the people they care for based on inference and critical thinking strategies, based on their experiences, knowledge, values, intellectual aptitude, objectivity, intuition so that they have accurate decision-making.

### Study limitations

There was a predominance of specialists from the southeastern region of the country, which may have limited the analyses from different epidemiological, cultural, and clinical contexts.

### Contributions to nursing, health or public policy

This research, in addition to providing reflections on integralist and modern theories, which are fundamental for the identification of the ND under study, also provided the refinement of the “Imbalanced Energy Field” diagnosis elements, which was published in the NANDA-I 2024-2026 taxonomy^(26)^.

## CONCLUSIONS

The NANDA-I “Imbalanced Energy Field” diagnosis content analysis carried out by judges contributed to the revision and alteration of its elements so that it can be effectively used in clinical practice and thus contribute to comprehensive human care.

Clinical validity studies with different populations are needed to confirm the findings of content analysis and investigate the causal relationships between DCs and RFs validated in this study. In addition, new studies can contribute to the validity and identification of the other elements of the ND under study (populations at risk and associated conditions).

## Data Availability

The research data are available within the article.
