# Supplementary material for: Validity Study of the “Imbalanced Energy Field” Nursing Diagnosis
Source: Rev Bras Enferm. 2026 Feb 23;79:e20250119. doi: 10.1590/0034-7167-2025-0119 (PMC12933929; doi:10.1590/0034-7167-2025-0119)
Supplement: Translation

# Translation: Estudo de Validação do Diagnóstico de Enfermagem “Campo de Energia Desequilibrado”

**DOI:** 10.1590/0034-7167-2025-0119pt

**Authors:** Camila de Souza Carneiro, Ana Cristina de Sá, Gisele Saraiva Bispo Hirano, Viviane Martins Silva, Alba Lucia Bottura Leite de Barros

**Affiliations:** I Universidade Federal de São Paulo, Hospital São Paulo. São Paulo, São Paulo, Brasil.; II Therapeutic Touch International Association. São Paulo, São Paulo, Brasil.; III Centro Universitário das Faculdades Metropolitanas Unidas. São Paulo, São Paulo, Brasil.; IV Universidade Federal do Ceará. Fortaleza, Ceará, Brasil.; V Universidade Federal de São Paulo. São Paulo, São Paulo, Brasil.

**Keywords:** Diagnósticos de Enfermagem, Estudo de Validação, Técnica Delphi, Terminologia Padronizada em Enfermagem, Terapias Energéticas

## Abstract

**Objetivos:**

analisar o conteúdo do diagnóstico de enfermagem “Campo de Energia Desequilibrado” (00273).

**Métodos:**

estudo de validação de conteúdo de diagnóstico de enfermagem. Doze juízes avaliaram a relevância dos elementos do diagnóstico de enfermagem da NANDA-I “Campo de Energia Desequilibrado, além da clareza e precisão de suas definições conceituais e operacionais. Foi considerado adequado um Índice de Validade de Conteúdo ≥0,80.

**Resultados:**

todas as definições foram analisadas pelos juízes, além da validação das 22 características definidoras e cinco fatores relacionados já existentes na versão NANDA-I 2021–2023. Foram sugeridas a exclusão de sete características definidoras e alteração de termos de dez (Índice de Concordância-Delphi ≥ 80%). As definições conceituais e operacionais dessas características definidoras foram construídas, validando-as pelo Índice de Validade de Conteúdo ≥0,80.

**Conclusões:**

após a validação, 15 características definidoras e cinco fatores relacionados foram julgados relevantes, e as alterações sugeridas foram aprovadas e publicadas pela NANDA-I 2024-2026.

## INTRODUÇÃO

Os diagnósticos de enfermagem (DEs) descritos na taxonomia NANDA-I têm a finalidade de fornecer embasamento científico à prática do cuidar direcionado ao plano terapêutico focado em intervenções de enfermagem acuradas^(1)^.

O DE “Campo de Energia Perturbado” (00050) foi inserido na NANDA-I em 1994 com a definição: “Distúrbio do fluxo de energia que envolve uma pessoa, resultando em desarmonia do corpo, mente e/ou espírito”. Em 2011, devido à falta de evidências para este diagnóstico e à sua semelhança com a intervenção “Toque Terapêutico” (5465) da *Nursing Interventions Classification^(2)^*, a NANDA-I decidiu revisá-lo baseando-se em estudos de revisões conceituais, análise de conceito dos seus elementos diagnósticos e estudo qualitativo, que utilizou questionários *online* com 426 enfermeiros holísticos que o utilizavam quando aplicavam técnicas energéticas^(3,4)^.

A partir dessas pesquisas, o DE “Campo de Energia Desequilibrado” (00273) foi inserido na taxonomia NANDA-I 2018-2020^(1)^ e foi modificado na edição NANDA-I 2021-2023^(5)^, embora ainda não tenha sido validado para sua efetiva aplicabilidade na prática clínica. *A priori*, identifica-se que, nos seus fatores relacionados (FRs), predominam transtornos emocionais como ansiedade e estresse excessivo. Fatores relacionados ao estresse afetam 90% da população mundial, e estão intimamente ligados às doenças do aparelho circulatório. Estresse e ansiedade ameaçam o equilíbrio e bem-estar do indivíduo, ocasionando alterações somáticas físicas como gastrite, hipertensão arterial, diabetes mellitus, entre outras comorbidades^(6–8)^.

Estudo prévio desenvolveu as definições conceituais e operacionais para as características definidoras (CDs) e FRs do referido DE, pois além de ser a primeira etapa de estudo de validação diagnóstica, este diagnóstico possui os termos “campo de energia” e “fluxo de energia”, que ainda são muito criticados pela ciência biomédica e necessitavam ser conceituados para facilitar sua aplicabilidade clínica^(9)^.

## OBJETIVOS

Analisar o conteúdo do diagnóstico “Campo de Energia Desequilibrado”, de modo a contribuir para o desenvolvimento de uma estrutura diagnóstica mais consistente que permita um melhor suporte à prática clínica de enfermagem, a partir do processo de inferência diagnóstica do enfermeiro.

## MÉTODOS

Foi realizado estudo de validação de conteúdo de um DE segundo as etapas modificadas propostas por Hoskins^(10)^. De acordo com este autor, o processo de validação deve ocorrer em três etapas: revisão integrativa; validação de conteúdo das CDs e FRs em estudo com especialistas; e validação clínica das definições conceituais e operacionais das CDs e FRs do DE. A primeira etapa constitui-se em revisão integrativa para definir conceitual e operacionalmente as CDs e FRs do diagnóstico “Campo de Energia Desequilibrado”^(9)^, sendo que o presente estudo trata da segunda etapa (validação das CDs e FRs com especialistas), que ocorreu no período de junho a outubro de 2022.

### Definição da amostra

A amostra foi composta por enfermeiros com experiência acadêmica e clínica com DEs e/ou Práticas Integrativas e Complementares em Saúde (PICS).

Os profissionais foram selecionados, inicialmente, com base nos critérios mínimos estabelecidos pelos pesquisadores como: experiência prática com DEs e/ou PICS; experiência acadêmica; e participação em grupos de pesquisa sobre a temática. Seu nível de *expertise* foi definido de acordo com a pontuação obtida: escore mínimo 05 pontos - especialista júnior; escore entre 05 e 11 pontos - especialista máster; escore máximo 11 pontos - especialista sênior^(11)^.

Os dados dos especialistas foram coletados diretamente na Plataforma *Lattes* e no Diretório do Grupo de Pesquisas do Conselho Nacional de Desenvolvimento Científico e Tecnológico. O convite, feito via e-mail, incluiu a descrição dos objetivos do estudo e o Termo de Consentimento Livre e Esclarecido (TCLE). Após o aceite e assinatura do TCLE, os especialistas selecionados receberam um formulário eletrônico com questões sobre seu perfil, caracterização sociodemográfica, definições conceituais e operacionais das 22 CDs, e os cinco FRs descritos na NANDA-I 2021-2023. Os avaliadores atribuíram um valor quanto à relevância das 22 CDs e dos cinco FRs, bem como a precisão e clareza de suas respectivas definições conceituais e operacionais, utilizando escala tipo Likert de 0 a 2, sendo: 0 – inadequado; 1 - pouco adequado; e 2 - adequado. Foram considerados adequados os itens que obtiveram concordância igual ou superior a 80% entre os juízes.

O tamanho da amostra de juízes em estudos de validação de conteúdo pela técnica Delphi tem sido um grande obstáculo. Embora para a utilização desta técnica não exista uma definição de quantidade mínima de especialistas, para este estudo, estabeleceu-se um mínimo de dez, conforme apontado pela literatura^(12)^.

O convite para participação desta etapa da pesquisa foi enviado por e-mail a 20 enfermeiros, considerando a dificuldade previamente documentada em estudos semelhantes quanto à taxa de retorno de formulários preenchidos em tempo hábil^(13)^. A amostra totalizou 12 especialistas na primeira rodada, 11 especialistas na segunda rodada e dez especialistas na terceira rodada de avaliação.

### Análise e tratamento de dados

Os dados da caracterização dos especialistas foram analisados segundo as frequências absolutas (f) e relativas (%). Os itens do instrumento que alcançaram consenso entre os especialistas de 80% ou mais foram considerados necessários e foram mantidos no instrumento de coleta de dados. As sugestões dos participantes consideradas pertinentes foram incluídas e enviadas para novo julgamento dos especialistas. Foram calculados a proporção e o Intervalo de Confiança de 95% (IC95%) de todos os elementos diagnósticos, além de suas definições conceituais e operacionais.

### Aspectos éticos

O estudo foi conduzido de acordo com as diretrizes de ética nacionais e internacionais, e foi aprovado pelo Comitê de Ética em Pesquisa da Universidade Federal de São Paulo (Certificado de Apresentação para Apreciação Ética nº 29110620.0.0000.5505), cujo parecer está anexado à presente submissão. Todos os juízes assinaram um TCLE, e o anonimato foi garantido.

## RESULTADOS

Todos os especialistas que participaram deste estudo eram enfermeiros (12). Houve predominância do sexo feminino (11), atuantes na região Sudeste do Brasil (cinco) e nas regiões Nordeste e Sul (três), respectivamente, com título máximo de doutorado (12), ocupação atual como docente e pesquisador (11), exercendo suas atividades profissionais em instituições de ensino (11).

Em relação ao uso do DE, nove juízes afirmaram que o utilizam tanto na prática clínica quanto no ensino. Dos juízes, nove lecionam disciplinas relacionadas ao DE, e 12 lecionavam sobre PICS. Quanto à idade dos juízes, sete encontram-se na faixa entre 30 e 45 anos, e cinco, entre 50 e 65 anos. A totalidade dos especialistas possui experiência profissional com as PICS acima de 12 anos, e oito participam de um grupo de pesquisa sobre a temática.

Para atingir um consenso mínimo de 80% para validação de conteúdo dos elementos do diagnóstico “Campo de Energia Desequilibrado”, foram necessárias três rodadas. Além do nível de concordância, foram calculadas a proporção e o IC95% das CDs e FRs, além de suas definições conceituais e operacionais. Os resultados da primeira rodada estão descritos na Tabela 1.

**Tabela 1:** Resultados da avaliação dos especialistas na primeira rodada de avaliação dos elementos diagnósticos e suas definições conceituais e operacionais (N = 12), São Paulo, São Paulo, Brasil, 2025

Itens	n	%	IC95% DC	n	%	IC95% DO	n	%	IC95%
CD - Padrões congestionados do campo de energia	9	75,0	42,8 – 93,3	7	58,3	28,5 – 83,5	7	58,3	28,5 – 83,5
CD - Padrões lentos do campo de energia	9	75,0	42,8 – 93,3	6	50,0	25,3 – 74,6	6	50,0	25,3 – 74,6
CD - Padrões rápidos do campo de energia	9	75,0	42,8 – 93,3	5	41,6	16,4 – 71,4	6	50,0	25,3 – 74,6
CD - Padrões fortes do campo de energia	10	83,3	50,8 – 97,0	5	41,6	16,4 – 71,4	6	50,0	25,3 – 74,6
CD - Padrões fracos do campo de energia	10	83,3	50,8 – 97,0	5	41,6	16,4 – 71,4	6	50,0	25,3 – 74,6
CD - Pulsações sentidas no fluxo energético	8	66,6	35,4 – 88,7	6	50,0	25,3 – 74,6	6	50,0	25,3 – 74,6
CD - Padrões tumultuados do campo de energia	6	50,0	25,3 – 74,6	5	41,6	16,4 – 71,4	6	50,0	25,3 – 74,6
CD - Ritmos dissonantes sentidos no fluxo energético	8	66,6	35,4 – 88,7	6	50,0	25,3 – 74,6	6	50,0	25,3 – 74,6
CD - Padrões de campo de energia com frequência que varia de pulsátil a latejante	7	58,3	28,5 – 83,5	5	41,6	16,4 – 71,4	6	50,0	25,3 – 74,6
CD - Padrões aleatórios do campo de energia	7	58,3	28,5 – 83,5	5	41,6	16,4 – 71,4	6	50,0	25,3 – 74,6
CD - Padrões irregulares do campo de energia	11	91,6	59,7 – 99,5	8	66,6	35,4 – 88,7	6	50,0	25,3 – 74,6
CD - Padrões de campo de energia arrítmicos	6	50,0	25,3 – 74,6	5	41,6	16,4 – 71,4	6	50,0	25,3 – 74,6
CD - Diferenciais de temperatura quente no fluxo energético	10	83,3	50,8 – 97,0	6	50,0	25,3 – 74,6	6	50,0	25,3 – 74,6
CD - Diferenciais de temperatura fria no fluxo energético	10	83,3	50,8 – 97,0	5	41,6	16,4 – 71,4	6	50,0	25,3 – 74,6
CD - Tração magnética para uma área do campo de energia	11	91,6	59,7 – 99,5	5	41,6	16,4 – 71,4	6	50,0	25,3 – 74,6
CD - Déficit de energia do fluxo energético	9	75,0	42,8 – 93,3	5	41,6	16,4 – 71,4	6	50,0	25,3 – 74,6
CD - Hiperatividade do fluxo energético	8	66,6	35,4 – 88,7	5	41,6	16,4 – 71,4	6	50,0	25,3 – 74,6
CD - Congestionamento do fluxo energético	8	66,6	35,4 – 88,7	7	58,3	28,5 – 83,5	6	50,0	25,3 – 74,6
CD - Bloqueio do fluxo energético	8	66,6	35,4 – 88,7	5	41,6	16,4 – 71,4	6	50,0	25,3 – 74,6
CD - Sensação de formigamento no fluxo energético	9	75,0	42,8 – 93,3	5	41,6	16,4 – 71,4	6	50,0	25,3 – 74,6
CD - Ritmos dissonantes dos padrões do campo de energia	6	50,0	25,3 – 74,6	6	50,0	25,3 – 74,6	7	58,3	28,5 – 83,5
CD - Expressão da necessidade de recuperar a experiência do todo	9	75,0	42,8 – 93,3	9	75,0	42,8 – 93,3	7	58,3	28,5 – 83,5
FR - Ansiedade	11	91,6	59,7 – 99,5	7	58,3	28,5 – 83,5	9	75,0	42,8 – 93,3
FR - Desconforto	12	100,0	69,8 – 100,0	10	83,3	50,8 – 97,0	8	66,6	35,4 – 88,7
FR - Intervenções que perturbam o fluxo ou o padrão energético	9	75,0	42,8 – 93,3	6	50,0	25,3 – 74,6	7	58,3	28,5 – 83,5
FR - Estresse excessivo	10	83,3	50,8 – 97,0	11	91,6	59,7 – 99,5	10	83,3	50,8 – 97,0
FR - Dor	12	100,0	69,8 – 100,0	10	83,3	50,8 – 97,0	10	83,3	50,8 – 97,0

*CD - característica definidora; FR - fator relacionado; DC - definição conceitual; DO - definição operacional; IC95% - Intervalo de Confiança de 95%*.

A partir da segunda rodada, além da avaliação da relevância das CDs e FRs, e clareza e precisão das definições conceituais e operacionais desses elementos diagnósticos, também foram analisados os significados e a semântica dos termos das CDs e FRs. Neste momento, foram feitas sugestões para mudança de termos e até exclusão de algumas CDs. Estas modificações foram:

A CD “Padrões lentos do campo de energia” foi alterada para “Padrão lento de fluxo energético” (IC90,9%); a CD “Padrões rápidos do campo de energia” foi alterada para “Padrão rápido de fluxo energético” (IC90,9%); a CD “Padrões fortes do campo de energia” foi alterada para “Padrão de campo de energia aumentado ou alto” (IC100%); a CD “Padrões fracos do campo de energia” foi alterada para “Padrão de campo de energia baixo” (IC90,9%); a CD “Ritmos assincrônicos sentidos no fluxo energéticos” foi alterada para “Ritmos assincrônicos no fluxo energético” (IC81,8%); a CD “Diferenciais de temperatura quente no fluxo energético” foi alterada para “Diferenciais de temperatura quente no campo de energia ou no fluxo energético” (IC100%); a CD “Diferenciais de temperatura fria no fluxo energético” foi alterada para “Diferenciais de temperatura fria no campo de energia ou no fluxo energético” (IC100%); a CD “Tração magnética para uma área do campo de energia” foi alterada para “Tração para uma área do campo de energia” (IC90,9%); a CD “Expressão da necessidade de recuperar a experiência do todo” foi alterada para “Expressão de sentir-se desintegrado, desmotivado ou desajustado” (IC90,9%); e o FR “Intervenções que perturbam o fluxo ou o padrão energético” foi alterado para “Tratamentos e/ou procedimentos em saúde que perturbam o fluxo ou o padrão energético” (IC81,8%).

As CDs “Padrões aleatórios do campo de energia” e “Sensação de formigamento no fluxo energético” foram excluídas na terceira rodada, tendo em vista que o seu conceito e a sua operacionalização já estão contemplados em outras CDs (IC80% e IC100%). A CD “Hiperatividade do fluxo energético” foi excluída, uma vez que a literatura não descreve este evento e os especialistas também o desconhecem (IC100%); a CD “Padrões tumultuados do campo de energia” foi excluída pois seus termos são sinônimos da CD “Padrões irregulares do campo de energia” (IC100%); a CD “Congestionamento do fluxo energético” foi excluída pois a CD “Padrões congestionados do campo de energia” já identifica congestionamento energético (IC90,9%); a CD “Ritmos dissonantes dos padrões do campo de energia” foi excluída, tendo em vista que seus termos são sinônimos da CD “Padrões tumultuados do campo de energia” (IC100%); e a CD “Padrões de campo de energia arrítmicos” foi excluída pois não se identifica “arritimicidade” do campo de energia na literatura e nem na prática clínica de especialistas (IC100%).

Os resultados do consenso entre os especialistas, a proporção e o IC95% da segunda rodada estão detalhados na Tabela 2, com exceção dos itens que foram excluídos do instrumento.

**Tabela 2:** Resultados da avaliação dos especialistas na segunda rodada de avaliação dos elementos diagnósticos e suas definições conceituais e operacionais (N = 11), São Paulo, São Paulo, Brasil, 2025

Itens	n	%	IC95% DC	n	%	IC95% DO	n	%	IC95%
CD - Padrões congestionados do campo de energia	10	90,9	57,1 – 99,5	10	90,9	57,1 – 99,5	10	90,9	57,1 – 99,5
CD - Padrão lento de fluxo energético	10	90,9	57,1 – 99,5	9	81,8	47,7 – 96,7	9	81,8	47,7 – 96,7
CD - Padrão rápido de fluxo energético	10	90,9	57,1 – 99,5	9	81,8	47,7 – 96,7	9	81,8	47,7 – 96,7
CD - Padrão de campo de energia aumentado ou alto	11	100,0	67,8 – 100,0	10	90,9	57,1 – 99,5	10	90,9	57,1 – 99,5
CD - Padrão de campo de energia baixo	10	90,9	57,1 – 99,5	8	72,7	39,3 – 92,6	9	81,8	47,7 – 96,7
CD - Pulsações sentidas no fluxo energético	11	100,0	67,8 – 100,0	11	100,0	67,8 – 100,0	9	81,8	47,7 – 96,7
CD - Padrões irregulares do campo de energia	11	100,0	67,8 – 100,0	9	81,8	47,7 – 96,7	9	81,8	47,7 – 96,7
CD - Ritmos assincrônicos no fluxo energético	9	81,8	47,7 – 96,7	9	81,8	47,7 – 96,7	7	63,6	31,6 – 87,6
CD - Padrões de campo de energia com frequência que varia de pulsátil a latejante	10	90,9	57,1 – 99,5	10	90,9	57,1 – 99,5	8	72,7	39,3 – 92,6
CD - Padrões aleatórios do campo de energia	8	72,7	39,3 – 92,6	8	72,7	39,3 – 92,6	9	81,8	47,7 – 96,7
CD - Diferenciais de temperatura quente no campo de energia ou no fluxo energético	11	100,0	67,8 – 100,0	10	90,9	57,1 – 99,5	8	72,7	39,3 – 92,6
CD - Diferenciais de temperatura fria no campo de energia ou no fluxo energético	11	100,0	67,8 – 100,0	11	100,0	67,8 – 100,0	10	90,9	57,1 – 99,5
CD - Tração magnética para uma área do campo de energia	10	90,9	57,1 – 99,5	11	100,0	67,8 – 100,0	10	90,9	57,1 – 99,5
CD - Déficit de energia do fluxo energético	10	90,9	57,1 – 99,5	9	81,8	47,7 – 96,7	8	72,7	39,3 – 92,6
CD - Bloqueio do fluxo energético	9	81,8	47,7 – 96,7	8	81,8	47,7 – 96,7	8	72,7	39,3 – 92,6
CD - Sensação de formigamento no fluxo energético	8	72,7	39,3 – 92,6	8	72,7	39,3 – 92,6	7	63,6	31,6 – 87,6
CD - Expressão de sentir-se desintegrado, desmotivado ou desajustado	10	90,9	57,1 – 99,5	10	90,9	57,1 – 99,5	9	81,8	47,7 – 96,7
CD - Hiperatividade do fluxo energético	8	72,7	39,3 – 92,6	8	72,7	39,3 – 92,6	7	63,6	31,6 – 87,6
CD - Padrões tumultuados do campo de energia	7	63,6	31,6 – 87,6	7	63,6	31,6 – 87,6	8	72,7	39,3 – 92,6
CD - Ritmos dissonantes dos padrões do campo de energia	9	81,8	47,7 – 96,7	7	63,6	31,6 – 87,6	7	63,6	31,6 – 87,6
CD - Congestionamento do fluxo energético	7	63,6	31,6 – 87,6	8	72,7	39,3 – 92,6	9	81,8	47,7 – 96,7
CD - Padrões de campo de energia arrítmicos	7	63,6	31,6 – 87,6	7	63,6	31,6 – 87,6	7	63,6	31,6 – 87,6
FR - Ansiedade	9	81,8	47,7 – 96,7	10	90,9	57,1 – 99,5	11	100,0	67,8 – 100,0
FR - Desconforto	11	100,0	67,8 – 100,0				8	72,7	39,3 – 92,6
FR - Tratamentos e/ou procedimentos em saúde que perturbam o fluxo ou o padrão energético	11	100,0	67,8 – 100,0	9	81,8	47,7 – 96,7	10	90,9	57,1 – 99,5
FR - Dor	11	100,0	67,8 – 100,0	10	90,9	57,1 – 99,5			

*CD - característica definidora; FR - fator relacionado; DC - definição conceitual; DO - definição operacional; IC95% - Intervalo de Confiança de 95%*.

A avaliação da terceira rodada dos elementos diagnósticos e suas definições conceituais e operacionais, com exceção dos itens que foram suprimidos do instrumento final, atingiu consenso acima de 80%. Seguem: a definição conceitual da CD “Padrões de campo de energia baixo”; a definição operacional da CD “Ritmos assincrônicos no fluxo energético”; a definição operacional da CD “Padrões de campo de energia com frequência que varia de pulsátil a latejante”; a definição operacional da CD “Diferenciais de temperatura quente no campo de energia ou no fluxo energético”; a definição operacional da CD “Déficit de energia do fluxo energético”; a definição operacional da CD “Bloqueio do fluxo energético”.

Devido à grande extensão das definições conceituais e operacionais validadas por este estudo, será apresentado a seguir somente um modelo (Quadro 1).

**Quadro 1:** Definições conceituais e operacionais elaboradas para as características definidoras e fatores relacionados do diagnóstico “Campo de Energia Desequilibrado”, São Paulo, São Paulo, Brasil, 2025

*Características definidoras*	*Definição conceitual*	*Definição operacional*
Padrões congestionados do campo de energia^(3,4,9,14–18)^	Percepção sensorial de congestão do campo de energia, com abaulamento do campo imediatamente anterior à interrupção parcial ou total do campo de energia e do fluxo energético, acompanhado de percepção sensorial de contrapressão, adormecimento e/ou choque.	O procedimento deve ser explicado ao participante e, em seguida, o profissional deve: (1) centrar-se conscientemente, focando no participante, e, assim, ativar um estado de percepção estendida; (2) avaliar o campo energético, mantendo as mãos a uma distância entre 5 e 10 cm com margem de diferença de 2 a 4 cm para mais ou para menos do corpo do paciente, mantendo-as na tangência entre o campo de energia humano e campo de energia ambiental, para explorar congestionamentos no campo de energia, que gera abaulamento do mesmo, déficits do fluxo de energia e energia desequilibrada; (3) tratar as áreas afetadas e modular o campo humano, para direcionar e equilibrar a energia em sua dimensão e simetria, com o objetivo de promover o livre fluxo de energia e harmonização do campo humano como um todo; nos casos de congestão e contrapressão, poderá ser necessário realizar, além do movimento de alisamento, o de desbloqueamento do campo para restabelecer o fluxo na área imediatamente após a área de congestão na direção céfalopodal; e (4) avaliar o campo energético e finalizar o tratamento.
***Fatores relacionados***	***Definições conceituais***	***Definições operacionais***
Ansiedade^(3,4,7,9,14–18)^	Sinais e sintomas de estresse; angústia; preocupações exageradas com saúde, dinheiro, família ou trabalho; tensões ou medo extremo de algum objeto ou situação em particular; medo exagerado de ser humilhado; sensação contínua de que um desastre ou algo muito ruim vai acontecer; falta de controle sobre os pensamentos, imagens ou atitudes, que se repetem independentemente da vontade; pavor depois de uma situação muito difícil.	Aplicar a *Hospital Anxiety and Depression Scale* para avaliação do nível de ansiedade e depressão.

Esta pesquisa necessitou de três rodadas para aprimoramento dos elementos do diagnóstico “Campo de Energia Desequilibrado” e suas definições conceituais e operacionais, que foram submetidos à NANDA-I para o seu aprimoramento e melhor aplicabilidade na prática clínica.

## DISCUSSÃO

As definições conceituais e operacionais das CDs e FRs dos DEs são primordiais para validar o seu conteúdo, pois estabelece descrições observacionais e práticas para aprimoramento e aplicabilidade clínica do diagnóstico^(14)^.

Ademais, podem servir para aumentar a confiabilidade e validade de dados clínicos relacionados aos diagnósticos. Além disso, podem, dessa maneira, favorecer a replicação de pesquisa, melhorar a capacidade do pesquisador de correlacionar a descoberta de estudos anteriores, e possibilitar a construção de indicadores para escolha das intervenções de enfermagem e avaliações dos resultados^(15–19)^.

As CDs “Padrões lentos do campo de energia”, “Padrões rápidos do campo de energia”, “Diferenciais de temperatura quente no fluxo energético” e “Diferenciais de temperatura fria no fluxo energético” foram alteradas, visto que as pesquisadoras e os especialistas, durante as etapas de validação, identificaram que os termos “campo de energia” e “fluxo de energia” foram descritos como sinônimos nas CDs. Entretanto, os seus conceitos, constituições psíquicas e energéticas são diferentes. As CDs “Padrões aleatórios do campo de energia”, “Sensação de formigamento no fluxo energético”, “Hiperatividade do fluxo energético”, “Padrões tumultuados do campo de energia”, “Padrões irregulares do campo de energia”, “Congestionamento do fluxo energético”, “Ritmos dissonantes dos padrões do campo de energia” e “Padrões de campo de energia arrítmicos” também foram excluídas pois os especialistas identificaram que estas não tinham evidências na literatura e não eram utilizadas na prática clínica, e também os termos eram repetitivos e não representavam efetivamente indicadores clínicos. Já o FR “Intervenções que perturbam o fluxo ou o padrão energético” foi alterado para “Tratamentos e/ou procedimentos em saúde que perturbam o fluxo ou o padrão energético”, visto que os termos modificados são mais claros e precisos. A validação de conteúdo deste DE foi acurada para que este possa realmente ser utilizado na prática clínica com segurança e sem dúvidas quanto aos significados e relevância de seus elementos. O campo de energia humano ou aura é a parte da energia universal associada ao corpo físico, e os *chakras* são os vórtices que movimentam e metabolizam a energia do campo de energia universal para o campo de energia humano. Ou seja, os *chakras* são aberturas por onde entra e sai o fluxo de energia da aura, e as doenças são geralmente causadas por uma obstrução ou interrupção do fluxo de energia^(20–24)^.

É bem conhecido que tensão, mau humor, pensamentos negativos e decisões impróprias no cotidiano levam os indivíduos a um estado de sobrecarga física e emocional, resultando em fadiga e outros desequilíbrios que podem exaurir as funções orgânicas. O corpo humano, sendo altamente eficiente e contando com considerável reserva funcional, normalmente se adapta a essas sobrecargas. No entanto, quando o estresse se torna excessivo, tal adaptação deixa de ocorrer, gerando a somatização de doenças em áreas do corpo que já são vulneráveis devido a fatores hereditários ou predisposições genéticas. Exemplos desses problemas incluem gastrite, colite, insônia, dor de cabeça, tonturas, resfriados recorrentes, dores cervicais ou lombares, diarreia e palpitações^(20–25)^.

Ainda sob essa perspectiva, foi também sugerido à taxonomia NANDA-I que houvesse alteração de alguns termos na definição do diagnóstico “Campo de Energia Desequilibrado” para adequação semântica, bem como adequação ao conceito de campo de energia humano (campo eletromagnético humano), que difere de fluxo de energia, conforme descrito acima. Por isso, a definição do diagnóstico em análise foi alterada de “Ruptura no fluxo vital de energia humana, que costuma ser um todo contínuo único, dinâmico, criativo e não linear” para “Uma interrupção no campo eletromagnético humano que costuma ser um todo contínuo e único, dinâmico, criativo e não linear”.

Ademais, foi sugerido que o domínio e a classe do diagnóstico “Campo de Energia Desequilibrado” fossem alterados, devido a fundamentos de teorias humanísticas e holísticas^(25–27)^ e paradigmas da saúde coletiva. Tais sugestões foram encaminhadas para o comitê de DEs da NANDA-I, o que levou à mudança de alocação do diagnóstico “Campo de Energia Desequilibrado” em um outro domínio e classe na edição de 2024-2026 (Domínio 1 - Promoção da Saúde; e Classe 1 – Percepção da Saúde)^(26)^.

Partindo da concepção ampla do processo de saúde-doença e de seus determinantes, a promoção à saúde propõe a articulação de saberes técnicos e populares, além da aliança de recursos institucionais, comunitários, públicos e privados, favorecendo a qualidade de vida, solidariedade, equidade, democracia, cidadania e integração dos indivíduos nas terapêuticas com autonomia e empoderamento^(27)^.

O enfermeiro avalia informações das pessoas que cuidam a partir da inferência e de estratégias de pensamento crítico com base em suas experiências, conhecimentos, valores, aptidão intelectual, objetividade e intuição, para que tenha uma tomada de decisão acurada.

### Limitações do estudo

Houve predominância de especialistas da região Sudeste do país, o que pode ter limitado as análises a partir de diferentes contextos epidemiológicos, culturais e clínicos.

### Contribuições para a área da enfermagem, saúde ou política pública

Esta pesquisa, além de fornecer reflexões sobre teorias integralistas e modernas, que são fundamentais para a identificação do DE em estudo, proporcionou o refinamento dos elementos do diagnóstico “Campo de Energia Desequilibrado”, que foi publicado na taxonomia NANDA-I 2024-2026.

## CONCLUSÕES

A análise de conteúdo do diagnóstico NANDA-I “Campo de Energia Desequilibrado” realizada pelos juízes contribuiu para revisão e alteração dos seus elementos para que efetivamente seja utilizado na prática clínica e, assim, contribuir com um cuidado humano integral.

Estudos de validação clínica com diferentes populações são necessários para confirmar os achados da análise de conteúdo e investigar as relações causais entre CDs e FRs validados neste estudo. Ademais, novos estudos podem contribuir na validação e identificação dos demais elementos do DE em estudo (populações em risco e condições associadas).

## Data Availability

Os dados da pesquisa estão disponíveis no corpo do artigo.
